# The enfacement illusion in autism spectrum disorder: How interpersonal multisensory stimulation influences facial recognition of the self

**DOI:** 10.3389/fpsyt.2022.946066

**Published:** 2022-11-03

**Authors:** Nicolas Deltort, Joël Swendsen, Manuel Bouvard, Jean-René Cazalets, Anouck Amestoy

**Affiliations:** ^1^University of Bordeaux, CNRS, Aquitaine Institute for Cognitive and Integrative Neuroscience, INCIA, UMR 5287, Bordeaux, France; ^2^Centre hospitalier Charles-Perrens, Pôle universitaire de psychiatrie de l'enfant et de l'adolescent, Bordeaux, France; ^3^Ecole Pratique des Hautes Etudes, PSL Research University, Paris, France

**Keywords:** face recognition, autism spectrum disorders, identity, self-awareness, self, interpersonal multimodal sensory stimulation

## Abstract

At its most basic level, the sense of self is built upon awareness of one's body and the face holds special significance as the individual's most important and distinctive physical feature. Multimodal sensory integration is pivotal to experiencing one's own body as a coherent visual “self” representation is formed and maintained by matching felt and observed sensorimotor experiences in the mirror. While difficulties in individual facial identity recognition and in both self-referential cognition and empathy are frequently reported in individuals with autism spectrum disorder (ASD), studying the effect of multimodal sensory stimulation in this population is of relevant interest. The present study investigates for the first time the specific effect on Interpersonal Multisensory Stimulation (IMS) on face self-recognition in a sample of 30 adults with (*n* = 15) and without (*n* = 15) ASD, matched on age and sex. The results demonstrate atypical self-face recognition and absence of IMS effects (enfacement illusion) in adults with ASD compared to controls, indicating that multisensory integration failed in updating cognitive representations of one's own face among persons with this disorder. The results are discussed in the light of other findings indicating alterations in body enfacement illusion and automatic imitation in ASD as well as in the context of the theories of procedural perception and multisensory integration alterations.

## Introduction

Autism Spectrum Disorders (ASD) are lifelong pervasive neurodevelopmental disorders that result in difficulties in social communication and interaction, as well as sensory abnormalities, stereotypic repetitive behavioral patterns and limited interests and activities (1). Studies conducted over the last 20 years on the social characteristics of individuals with autism have highlighted particularities related to facial processing in general, ranging from the early stages of facial perception and detection that include atypical patterns of gaze fixation (2) to impairments in facial categorization related to identity, sex, age, or familiarity that rely on both memory and recognition abilities (3–6). However, the literature on this subject has produced inconsistent findings (7–14) possibly due to heterogeneity of the sample studies (15), variation in experimental paradigms that included familiarity cofounds, different retention times of the memory trace related to facial identity, or to the nature of face stimuli that sometimes included non-facial features such as hair or clothes (4, 16). These contradictory results may have contributed to a global underestimation of facial recognition difficulties in persons with ASD (6, 13).

Facial discrimination allows us to recognize an individual from another (Individual Identity Recognition, or IIR) which is crucial for social interaction (5). This ability emerges at early stages of human development (17, 18) and it is challenged by both intrinsic (e.g., age, sex, facial expression) and extrinsic (e.g., luminosity, contexts) factors that benefit from plastic neuronal face representations (4, 19). Throughout normal development, individuals may experience various differences in their face IIR abilities (20). Moreover, some studies have reported that 2–3% of adults in the general population have severe difficulties in recognizing the identity of faces in everyday life, a phenomenon known as prosopagnosia and that has recently been linked to the oxytocin system (21–23).

Of particular interest among all faces known to a given individuals is one's own face (self-recognition). For Lewis (24), self-recognition in a mirror would have the same representational underpinnings as those required for “psychological” self-consciousness (25) and would be closely associated with the use of personal pronouns and pretend play in typically-developing children. The processing and recognition of one's own face in autism has been the subject of a limited number of investigations using classic or modified mirror tests and they have produced with contrasting results (26–28). Although many developmental markers of self–representation and self-awareness are delayed or absent in children with ASD, including Theory of Mind functions, empathy, imitation or autobiographical memory (29, 30), basic self-face recognition as tested by the mirror recognition task appears to be unaffected in children with this disorder once a mental age of 2 years is achieved (26–28).

Visual recognition of one's own face contributes directly to the recognition (awareness) of the bodily self as distinct from others (31) and to the basic sense of self which is crucial to build individual identity (19). Neuroimaging studies have demonstrated that different networks are involved when perceiving and analyzing one's own face compared to a familiar or unfamiliar face, including the right inferior fronto-parietal cortex and bilateral inferior occipitotemporal cortex regions (32–34). Furthermore, advances in the understanding of body awareness have led to a reconsideration of the question of one's own face and its unimodal representation. For example, recognition of one's own face is facilitated by prior exposure to one's own smell or to the sight of one's own name, which introduces the notion of plurimodality in the construction of recognition of one's own face, in contrast with familiar faces processing (35). In ASD, fMRI studies indicate that adults with this disorder show atypical brain activation patterns compared to non-ASD participants, with significantly less activation in the posterior cingulate cortex and the right insula, associated with self-representations/agency when judging the “photogenic character” of their own face (36). These findings suggest dysfunctions in the self-representation/self-awareness network at a basic perceptual level of cognitive functioning. Neuroimaging studies using discrimination tasks based on “morphed pictures” (37) or “morphing videos” (38) between both pictures of one's own face and that of others (stranger face, familiar or unfamiliar) report different activations in the right prefrontal system (37) or in the inferior frontal gyrus (38) between ASD and non-ASD children.

Multimodal sensory integration is essential to awareness of one's own body, and similar to the well-known “rubber hand illusion” (39, 40), it has been also shown that different sensory modalities may also affect self-face recognition processes. Platek et al. (35) showed a significant decrease in reaction time for the recognition of one's own face when the individual was previously exposed to their own smell or to the sight (or hearing) of their own name. This was not the case for the recognition of familiar faces when previously exposed to their odors or names. These results suggest a potentiation of access to representations of one's own face by the sensory multimodality. This notion of multimodality was also tested by the “Enfacement Illusion” paradigm (41, 42) inspired by the “Rubber Hand Illusion” paradigm. Participants were subjected to tactile stimulation at the level of the cheek while they watched the face of another person being touched at the same level of the cheek. The presence of synchronous visual and tactile stimuli affected the representation of one's own face in the same way that the Rubber Hand Illusion altered the representation of one's own body. The perception of similarity and attractiveness to the other was significantly increased with synchronous stimulation (41, 43). The authors hypothesized that interpersonal synchrony had an effect on social cognition (43, 44).

Although investigations to date using the Enfacement Illusion paradigm have been conducted almost exclusively in non-clinical samples, it may have particular relevance to understanding difficulties in self-perception in persons with ASD. Previous studies suggest that children with autism aged from 8 to 18 years old tested by a “ Rubber Hand Illusion” (RHI) paradigm, exhibited reduced timing or/and efficacy to integrate visual and tactile information compared to non-ASD children (44). Other more recent ones indicate that adults with ASD were markedly less susceptible to the Full Body Illusion (FBI), not exhibiting the illusory self-identification and self-location drift, tested in a full body illusion set up where participants wore a head-mounted display showing a view of their “virtual body” being stroked synchronously or asynchronously with respect to felt stroking on their back (45). The first objective of the present study is therefore to assess self-recognition performance of adults with this disorder compared to non-ASD adults during a self-other discrimination task based on morphing videos. The second objective is to evaluate the effect of IMS (i.e., enfacement illusion) which on self-recognition performance in both groups.

## Materials and methods

### Participants

Thirty adults participated in the present study including 15 individuals with typical development (TD) and 15 diagnosed with ASD. Adults with ASD were recruited from the Bordeaux Autism Resource Center, France, where they were evaluated relative to DSM-5 criteria evaluation (1), the Autism Diagnostic Interview-Revised (ADI-R) (46) and the 2^nd^ version of the Autism Diagnostic Observation Schedule-Generic (ADOS-G) (47). Only individuals without intellectual disabilities were eligible for participation and additional exclusion criteria included known neurological and visual disorders. Exclusion criteria for the non ASD group were intellectual disability and other known developmental, neurological, visual disorders. The two groups were matched for age and sex (Table 1). This investigation was approved by the regional ethics review board (Comité de Protection des Personnes Bordeaux/CPP N° 100038-80) and the national commission for numeric data and liberty (CNIL:commission nationale de l'informatique et des libertés).

**Table 1:** Participant profiles.

	**ASD**	**TD**
*N*	15	15
Mean IQV or ICV (WAIS)	105.13 ± 12.25	-
Male sex	15	15
Mean age; range	28.5 ± 11; 18–54	28.5 ± 10 (SD); 18–54
ADOS-2 total score	8.06 ± 2.3	-
ADI-R SI^*^ total sub-score	19.3 ± 2.2	-
ADI-R Co^**^ total sub-score	10.7 ± 2.3	-
ADI-R RBR^***^ total sub-score	5.3 ± 1.5	-

Mean values ± SD.

ADI-R 4to5-ever/diagnostic:

* Social Interaction (SI),

** Communication (Co),

*** RBR: Restricted and Repetitive Behaviors (RBR).

### Procedure and measures

**Figure 1:**
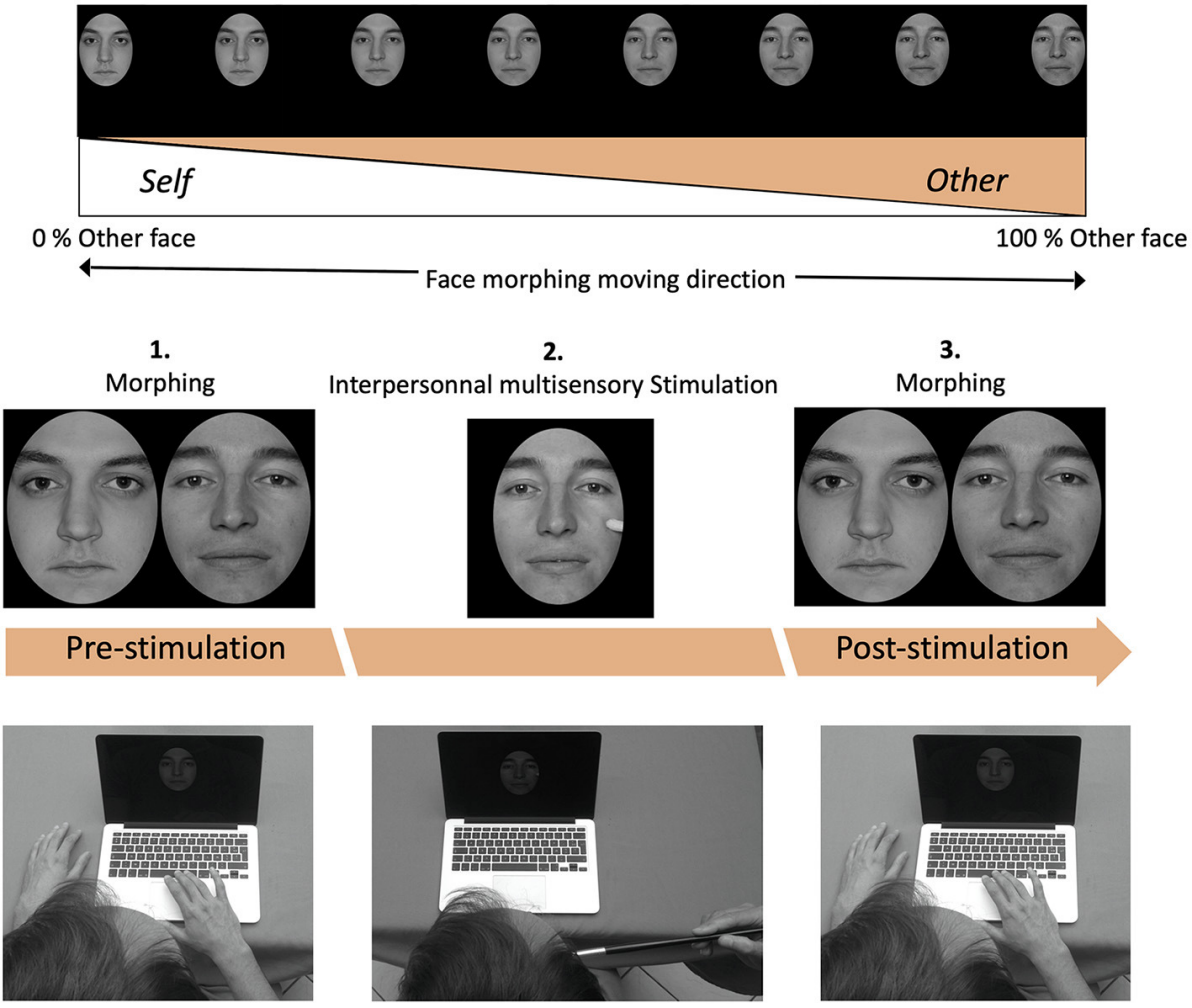
Experimental set-up of the self-recognition task with the two different morphing directions of the movies: from “self to other” (movie started from self-image and ended with other-image, or from “other to self” movie started from other-image and ended with self-image); Design of the experimental blocks containing three phases: pre-stimulation test, visual-tactile stimulation and post-stimulation test.

During the first inclusion visit, participants were informed of the study procedures and their rights before providing their informed written consent for the publication of any potentially identifiable data. A photograph of each participant's face with a neutral expression was then taken using a chinrest and a fixed focal-length camera placed at the same distance from the chinrest (112 cm). Before the experimental session, a computerized morphing procedure implementing a mesh warping algorithm (Morphage software, Creaceed, Belgium) was used to merge each participant's face with an unfamiliar face by 1% steps, resulting in a 100-frame movie with graded blending of the facial features of the two faces (Figure 1A).

Three unfamiliar male models (one for the participants' training session and two for the experimental session from a battery of male faces constructed for the study) were selected to create the morphing movie using a photograph taken under the same conditions as those of the participants (Figure 1A). This movie created for the interpersonal multisensory stimulation session was recorded with a neutral facial expression and with the model's head (maintained by a chinrest). During the video recording of 120 ms, the experimenter touched the model's right cheek with a brush for 2 s, every 2 s. Using After Effects software (Adobe, France) the photographs and movies were then converted to black and white (grayscale) format and the images were horizontally transposed in order to obtain a mirror image of the face. Non-facial attributes (hair, ears) were removed using a black template.

### Self vs. other face discrimination

During the pre-stimulation session (see Figure 1B for task chronology), participants first performed the face discrimination task by watching the morphing movie (Figure 1A) consisting of 100 frames. Each frame represented a 1% incremental change from one face to another, from ‘0 to 100% of Other's face content, in the “Self to Other” direction, or from 100 to 0% of Other's face content in the “Other to Self” direction. The participants were then asked to “click on the keyboard as soon as the image looks more like someone else than yourself” when the “Self to Other” direction was presented, and to “click on the keyboard as soon as the image looks more like yourself than someone else” when the “Other to Self” direction was presented. Self vs. Other face discrimination session consisted of 4 repetitions of the 2 conditions. The responses were expressed as a Percentage of Other Face content (POF) and they served as a baseline measure of self-face recognition performance of each participant before the IMS session.

### Interpersonal multisensory stimulation effect

Immediately after the pre-stimulation session, participants were administered the IMS session. In this experimental setting, IMS corresponds to a stimulation involving two senses (sight and touch). The participant looked at the screen and observed the movie that showed a paintbrush touching the same unfamiliar model's face that was also used during the pre-stimulation session. As soon as the movie appeared on the screen, tactile stimulation was delivered manually by the experimenter on a congruent location on participant's cheek with the standard round paintbrush during 2 s every 2 s (like in the movie they were watching), during 120 s (Figure 1). The experimenter listened through earphones to the audio file of the pre-recorded movie to pace the tactile stimulation in a synchronous or asynchronous tempo with the tactile stimulation watched by the participant. In the asynchronous condition, the asynchrony between visual and tactile stimulation was 1 sec.

The order of presentation of the 2 IMS conditions, synchronous or asynchronous, were randomized between participants. Therefore, while the participant was watching the film of an unfamiliar face being touched, he was being touched himself on the same facial location either at the same time (i.e., synchronous visual-tactile stimulation) or at different time onsets (i.e., asynchronous visual-tactile stimulation). The post-stimulation measure consisted of a second self-other discrimination task using morphing videos of the same direction as in the pre-stimulation session. The difference between the POF measured in the post-stimulation and the POF in the pre-stimulation sessions was used to evaluate the IMS effect i.e., enfacement.

As there were two morphing direction conditions (“Self to Other” and “Other to Self”) and two IMS conditions (synchronous and asynchronous), we crossed these conditions to have 4 experimental blocks which were repeated twice, each time with a different unfamiliar face. Therefore, eight experimental blocks differing in the choice of unfamiliar face, type of visuo-tactile stimulation (i.e., synchronous vs. asynchronous) and in the direction of morphing movie (i.e., “other to self” vs. “self to other”) were completed by each participant, and an order that was randomized across participants.

### Statistical analysis

POF values < 20% or > 80% were considered irrelevant and excluded from the analysis since they were related to attentional problems or impulsivity or errors. In total, this only concerned 6 values below 20% and 4 above 80% out of 240 responses. The “Delta-POF” was defined by the difference between the POF measured in the pre- and post-stimulation session. Statistical analyses were proceeded using IBM SPSS Statistics software (IBM Corporation, USA), and according to Laerd statistics recommendation (Laerd Statistics, London, UK). Values for all variables are expressed as means ± standard deviation (SD), unless otherwise specified. Residual analysis was carried out to test for ANOVA assumptions. Outliers were detected by visual inspection of box plots; normality was assessed using Shapiro-Wilk's normality test and homogeneity of variances by Levene's test. The level of significance was set at *p* = 0.05.

## Results

### Self vs. other face discrimination

**Figure 2:**
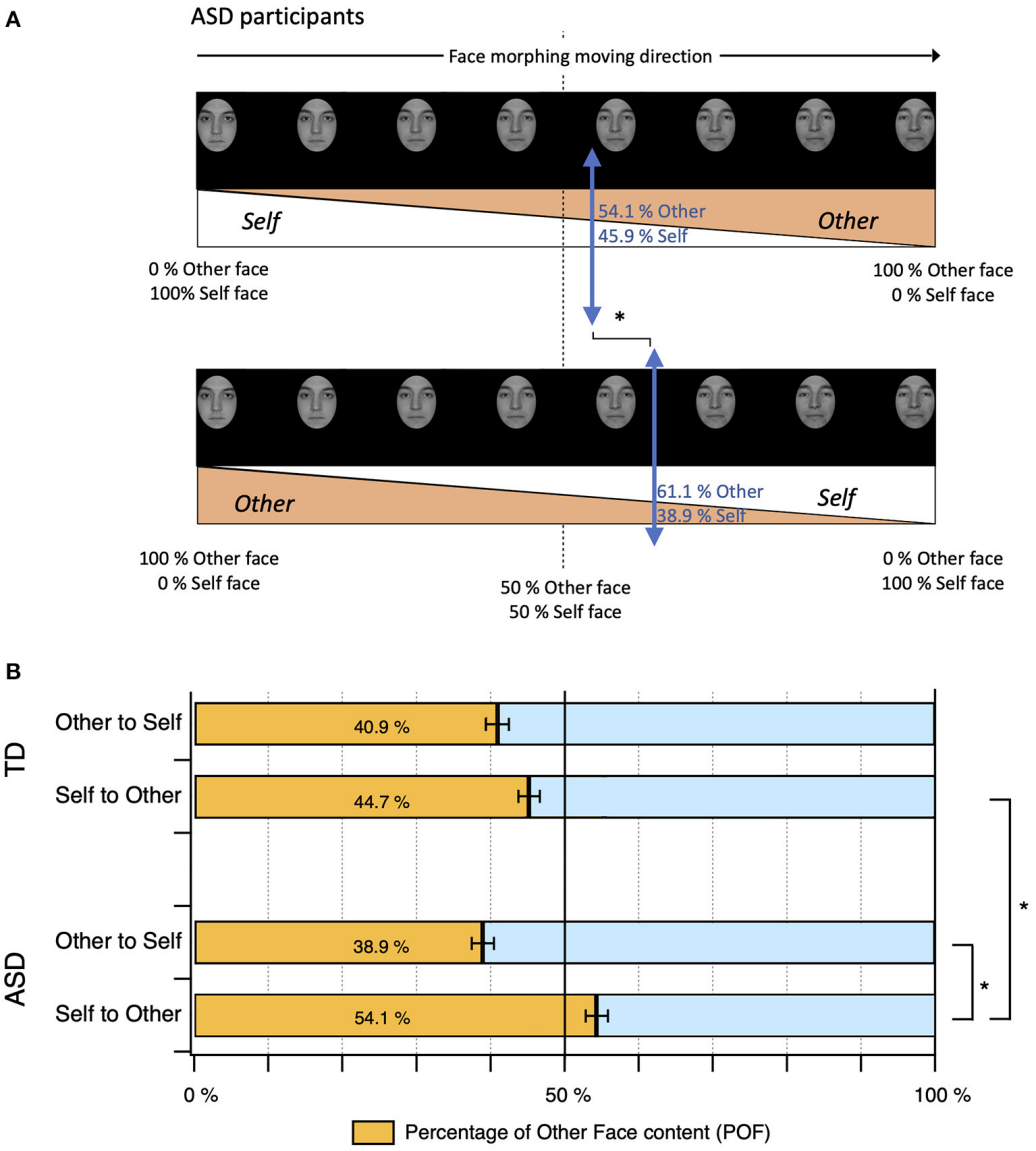
Discrimination of Self vs. Other face. **(A)** ASD participant responses during the task. **(B)** The bar graph presents the mean Percentage of Other Face (POF) content (orange bars) that participants perceived during the various conditions presented here.

Results from this experiment are presented in Figure 2 with Figure 2A highlighting ASD participant responses. In the Self to Other direction, ASD participants stopped the morphing sequence when the face contained 45.9% of Self face and 54.1% of Other face while in the Other to Self direction they stopped the video when it contained < 38.9% of unfamilliar faces (and 61.1% of Self). Figure 2B summarizes all the data. In the Self to Other direction, TD adults stopped the morphing movie when the image contained on average 44.6 % ± 1.7 (SEM) of the unfamiliar face, which was significantly different from ASD adults who stopped the morphing movie when it contained 54.1 % ± 1.5 (SEM) of the unfamiliar face. In the Other to Self direction, no differences were found between the two groups: TD participants stopped when the image contained 40.9 % ± 1.5 (SEM) of the unfamiliar face and ASD participants stopped when it contained 38.9 % ± 1.5 (SEM) of unfamiliar face (Figure 2).

We conducted a two-way ANOVA on the Percentage of Other Face content (POF) measured for each participant during the pre-stimulation sessions (Figure 2) with the direction of morphing (i.e., from Self to Other, or from Other to Self) as a within factor, and group (TD, ASD) as a between factor. No outliers were found by box plot inspection. Data were normally distributed as assessed by Shapiro-Wilk's test (*p* > 0.05), except for the ASD group in the direction ≪ Other to Self ≫ (*p* = 0.012). Two-way ANOVAs were conducted in light of their robustness to deviations from normality (48, 49). There was homogeneity of variances as assessed by Levene's test for equality of variances (*p* = 0.36). There was a statistically significant interaction between group and direction for POF, F(1, 220) = 14.78, *p* < 0.0001. We found a significant main effect of the direction of morphing, F(1, 220) = 51.51, *p* < 0.0001 in the ASD group, but not in the TD group. There was a statistically significant difference in mean POF between TD and ASD groups in the direction ≪ from Self to Other ≫, *F*(1, 220) = 20.68, *p* = 0.000 and the mean POF was 9.456 points higher (95% CI: 5.358–13.554) for the ASD group than for the TD group.

### Interpersonal multisensory stimulation effect

**Figure 3:**
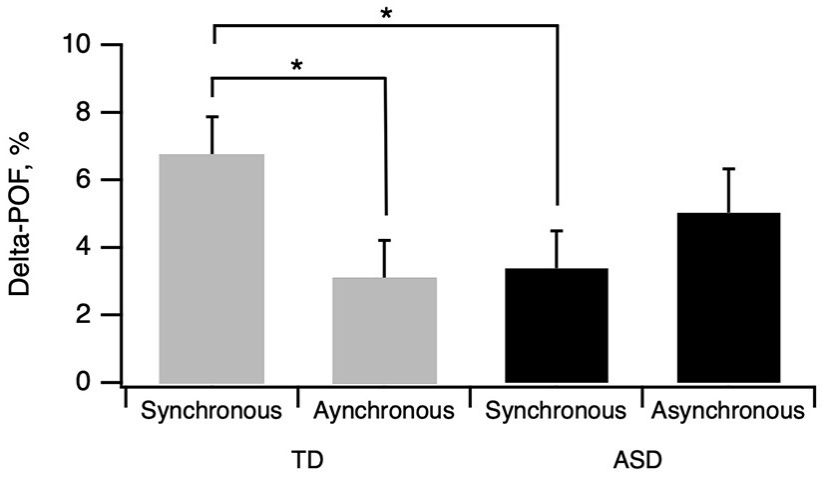
Effect of Interpersonal multisensory stimulation. Delta-POF was calculated as mean POF in poststimulation condition minus mean POF in prestimulation condition. POF: Percentage of Other Face. Asterisks indicate significant differences.

Subsequent to the stimulation sessions, the participants were tested again in the face recognition task. A Delta-POF value corresponding to the difference between the POF before and after the stimulation was computed (Figure 3). In the TD group, the mean Delta-POF for synchronous and asynchronous stimulation was 6.8 ± 1.1% (SEM) and 3.1 ± 1.1 (SEM), respectively (Figure 3). In the ASD group, the mean Delta-POF was 3.4 ± 1.2 and 5.03 ± 1.1 for synchronous and asynchronous stimulations respectively.

We conducted a three-way ANOVA on Delta-POF with the direction of morphing (i.e., from Self to Other, or from Other to Self) and stimulation (Synchronous or Asynchronous) as within factors and group (TD, ASD) as a between factor. Six outliers with Delta-POF values >1.5 box-lengths from the edge of the box plots were removed from analysis. Data were normally distributed as assessed by Shapiro-Wilk's test (*p* > 0.05). Although the assumption of homogeneity of variances was not respected as assessed by Levene's test for equality of variances, *p* = 0.007, three-way ANOVAs were conducted in light of their robustness to heterogeneity of variance in circumstances of approximately equality of group sample sizes (49).

There was no statistically significant three-way interactions between groups, stimulation and morphing direction, F(1, 203) = 0.024, p = 0.88. There was a statistically significant interaction between group and stimulation on Delta-POF, F(1, 203) = 5.51, *p* = 0.02. The stimulation had a statistically significant effect on Delta-POF for the TD group, *F*(1, 203) = 5.46, *p* = 0.02), but not for ASD group, *F*(1, 203) = 1.02, *p* = 0.32. There was a statistically significant effect of group on Delta-POF for the synchronous stimulation, (*F*(1, 203) = 4.03, *p* = 0.03, but not for the asynchronous stimulation, *F*(1, 203) = 1.46, *p* = 0.23. After the synchronous stimulation, the ASD group had a significantly lower mean Delta-POF than the TD group, −3.379 (95% CI, −6.54 to −0.22), *p* = 0.04. The simple main effect of direction on “Delta-POF” for ASD group was significant, F(1, 203) = 5.78, *p* = 0.017. ASD participants had a significantly higher mean Delta-POF for the Other to Self direction, than in the Self to Other direction, by 5.1 points (95% CI, 1.89–8.32), *p* = 0.002.

## Discussion

### Self vs. other discrimination

In agreement with previous studies, we found that in TD adults there was no difference in self-face discrimination according to the direction of the morphing movie and relative to the Percentage of Other Face (POF) content where these adults still recognized their “self” from an image that contained between 41 to 44,6% of “unfamiliar faces” (41, 42, 48, 49). In contrast, adults with ASD recognized as their “self” an image that contained up to 54 % of an unfamiliar face in the Self to Other direction, while they had similar discrimination behaviors to TD participants in the Other to Self direction. One explanation of face recognition deficits in ASD is that individuals with this disorder fail to develop normative levels of face-relevant perceptual expertise (10, 50). For TD individuals, increased experience with categories of face stimuli, such as human faces compared to the faces of other species (17, 51) or such as familiar faces compared to unfamiliar faces (52, 53) confers an expert level processing advantage for stimuli of that category (54), and more specifically for self faces. This advantage is believed to be related to the ability to integrate previously experienced examples into prototypic mental representations and to engage in configural processing (55). Unfamiliar faces are believed to engage analytical perceptual processing, in contrast with familiar and self faces that are thought to engage procedural processing. Individuals with ASD have failed to demonstrate many of these same makers (6, 56), with various studies suggesting atypical perceptual cue taking, atypical configural cognitive processing and atypical stored memory representation of familiar and unfamiliar faces (50, 57, 58). These particularities in face processing have been associated with the more global hypothesis of enhanced perceptual over-functioning in ASD as described by Enhanced Perceptual Function theory (EPF) (59).

This hypothesis suggests that all of the so-called “low-level” perceptual processes (discrimination, processing of psychophysical dimensions, encoding speed, perceptual matching) would be over-efficient in autism and would impose the persistence of the so-called “analytical” perceptual analysis of faces to the detriment of the installation of configural processing (13, 59, 60). According to this hypothesis, there is a bias in favor of analytical perceptual processing in people with ASD, which would explain the superiority of “local” perceptual processing in this disorder compared to those without ASD.

The typical focal and analytic processing used in the direction from Other to Self leads to performance in adults with ASD that is comparable to those of control subjects (again in the direction from Other to Self). However, this analytic processing would not compensate for the configural processing deficit believed to be used by non ASD individuals in the Self-to-Other condition when the movie started with the individual's own face, a difference which could explain the mismatch in this condition. In the Self to Other direction, participants with ASD likely proceeded with an analytical perception of the morphing video even when the video started from a pure image of self and they kept capturing all the “self-face components” that they could perceive. When they stopped the morphing video, the selected picture contains <46% of “self” and 54% of “unfamiliar face” (Figure 2A). In the Other to Self direction, video started with the unfamiliar face and with analytical processing again participants with autism kept capturing the unfamiliar components of the frames of the video. The selected picture contained at the time they stopped the video, <38% of unfamiliar faces when the judgment of “self” appeared to be preeminent (with analytical processing they needed higher quantity of “self-components” (62%) than when self-face was looking first in the Self to Other direction). One may speculate that this analytical processing is affected by a “first image priming effect” that lead to POF differentiation between both directions in ASD but not in TD adults. In contrast, TD participants, who are expected to rely on configural perceptual processing of their own faces, selected the same ratios of components of their own faces in both moving directions, with configural processing ensuring more stable holistic processing of faces that would be less affected by a “first frame priming effect.” This hypothesis needs findings to be replicated in larger samples and with various stimuli that are known to challenge and break down the configural processing in typically developed individuals as inverted face (61), composite faces or morphing objects (54, 62, 63). This hypothesis requires findings to be replicated in larger samples and with other stimuli known to challenge and break down the configural processing in typically developed individuals as inverted face (61), composite faces or morphing objects (54, 62, 63).

### Interpersonal multisensory stimulation effect

Consistent with past investigations, our results in TD adults showed that synchronous IMS changed self-other recognition performance by approximately 6% in the synchronous condition and 3% in the asynchronous condition relative to the baseline pre-test measure. Interpersonal multisensory stimulation (IMS) creates an enfacement illusion in individuals with typical development, but only in the synchronous condition (42, 43).

Synchronous IMS specifically affected recognition of the self-face, as statistically significant changes were observed only for the direction of morphing that presented a transition from other to self. When participants saw the face of the other being slowly morphed into the self-face, and were asked to indicate when the face looks more like themself, they stopped the movie significantly earlier compared to the pre-stimulation test. This pattern suggests that, following IMS, participants accepted as self-stimuli morphed faces that contained 6% more of the other's face. Importantly, no similar effects were observed for the reverse direction of morphing (i.e., “self to other”). This asymmetric effect for the two directions of morphing was also observed in previous studies using the same methodology using videos of unfamiliar faces during IMS (43) in contrast with previous studies using familiar faces (42, 49).

In our experiment, the IMS effect and enfacement illusion involved both visual and tactile perception and results from a change in what is expected for one of these stimuli. In particular, the visual feedback is modified by replacing one's own face with another face, while the tactile stimulus is consistent with what is seen and perceived when applied in a synchronous condition. Participants were asked to experience someone touching their face as they looked in the mirror, but what they saw in the mirror is not the face they consider to be theirs. The created illusion is, therefore, the development of the feeling that the face observed is belonging to the participant. That feeling is similar to that of the belonging of a rubber hand in the “rubber hand illusion”. Results reported that the process of identifying with a body seen in the mirror alters the processing of the visual stimuli applied to the reflected body, which are re-mapped as peri-personal stimuli through the mirror reflection (64). The synchronous condition that elicits the illusion results in multisensory driven predictions about upcoming somatosensory input. These predictions about the face are then constantly updated during multisensory experience, but only while exposed to a synchronic mirror reflection. These results support the hypothesis of neuronal plasticity of the self-face representation in typical development that account for the changes in the perceptual experience after synchronous IMS with the assimilation of features of the other's face in the mental representation of one's own face, as reported in previous studies investigating multisensory stimulation to the face (41, 65) and to the body (66). This plasticity ensures both the assimilation of changes and a sense of continuity overtime that is essential for the sense of identity, despite the fact that our appearance changes over time.

In adults with ASD, our findings for a lack of difference between synchronous and asynchronous conditions for the IMS effect, as well as a significant difference of performance compared with non-ASD adults in the synchronous condition, suggest an absence of the enfacement illusion in ASD. The enfacement illusion is based on IMS and requires efficacious multimodal integration processing. Yet difficulties in multimodal integration have previously been described in ASD relative to integration of audiovisual speech information [for e.g., see (67–69)] but also visuo-tactile information in two studies using the Rubber Hand Illusion (RHI) (44, 70), and two more recent ones based on the “Numbness Illusion” [NI, (71)] and on the “Full Body Illusion” (FBI, 45). As children with ASD did not exhibit the expected effect for rubber hand illusion in the synchronous brushing condition after 3 min, but did after 6 min, authors emphasized the impact of the timing of visual and tactile stimulation, suggesting that children with ASD would need a more extended temporal window to complete visuo-tactile binding (44, 72). To test this hypothesis further researches should design paradigms including several timings of stimulation in adult samples. These findings are coherent with previous results related to the embodiment theory that reported that the unconscious reproduction of the emotional facial expression by one's observer would be a mechanism for facilitating the recognition of the observed emotions, thus responsible for congruence between the stimulus and the facial muscle activations, facilitating emotional resonance and empathy. Related to the embodiment theory, the facial feedback hypothesis suggests that the experience of emotions in non-ASD individuals is affected by feedback from facial muscle activation (73). Stel et al. (74) demonstrated that automatic or voluntary facial expressions, modulated by holding a pen between the teeth, influenced corresponding emotions compared to non-pen holding in controls, while adolescents with ASD remained emotionally unaffected. These authors concluded that the facial feedback mechanism worked differently for individuals with ASD. Similarly, it is recognized that people with ASD may also present atypical mental representations of their own emotional experiences (75). A deficit in the multisensory integration (visual and proprioceptive) could, in ASD, partly explain impairment in self-face representations, but also difficulties in facial emotions expression, recognition, and resonance (mimicry) as it requires the individual to update our own self-face representations. ASD participants who were significantly less likely to experience the Rubber Hands Illusion or the Full Body Illusion, were participants who displayed less empathy in both studies with adults and children, and more severe autistic traits in the adult study only (44, 45). These results suggest that the altered bodily self-consciousness in ASD may contributes to social difficulties. Sensorimotor processes play an important role in the mentalization of one's internal states and intentions, and the present results may guide future investigations that test interventional paradigms to enhance multisensory integration.

## Conclusion

The present findings lend support to the hypothesis that neuronal plasticity during visual-tactile stimulation leads to another's face being perceived as one's own. The findings also support the conclusion that such a process that is essential for mirror self-recognition and provides dynamic representations of one's visual appearance and self-awareness, are impaired in ASD. These observations may be of key importance for understanding the neurobiological processes underlying the maintenance of a continuous sense of self and for understanding what is fundamentally different in persons with ASD. It seems essential to distinguish the processes of self-identification and self-recognition from the process of self-updating which differs specifically in ASD adults, thus supporting the hypothesis of impaired or atypical multisensory integration. Indeed the sample size of the current study is not so large, and it would be relevant in future studies to investigate in children, teenagers and females with ASD as well as in selected clinical subgroups that could be differentiated based on empathy scores severity or autistic traits.

## Data availability statement

The original contributions presented in the study are included in the article/supplementary material, further inquiries can be directed to the corresponding author.

## Ethics statement

The studies involving human participants were reviewed and approved by Bordeaux Regional Ethics Review Board (Comité de Protection des Personnes Bordeaux/CPP N° 100038-80). The patients/participants provided their written informed consent to participate in this study. Written informed consent was obtained from the participants for the publication of any potentially identifiable data or images included in this article.

## Author contributions

ND: experiment, analysis, conceptualization, and writing. JS: writing and editing. MB: conceptualization. J-RC: experiment, analysis, funding acquisition, supervision, writing, and editing. AA: funding acquisition, supervision, conceptualization, writing, and editing. All authors contributed to the article and approved the submitted version.

## Conflict of interest

The authors declare that the research was conducted in the absence of any commercial or financial relationships that could be construed as a potential conflict of interest.

## Publisher's note

All claims expressed in this article are solely those of the authors and do not necessarily represent those of their affiliated organizations, or those of the publisher, the editors and the reviewers. Any product that may be evaluated in this article, or claim that may be made by its manufacturer, is not guaranteed or endorsed by the publisher.
